# Discerning the Complexity of Community Interactions Using a *Drosophila* Model of Polymicrobial Infections

**DOI:** 10.1371/journal.ppat.1000184

**Published:** 2008-10-24

**Authors:** Christopher D. Sibley, Kangmin Duan, Carrie Fischer, Michael D. Parkins, Douglas G. Storey, Harvey R. Rabin, Michael G. Surette

**Affiliations:** 1 Department of Microbiology and Infectious Diseases, University of Calgary, Calgary, Alberta, Canada; 2 Molecular Microbiology Laboratory, Faculty of Life Sciences, Northwestern University, Xian, Shaanxi, China; 3 Department of Medicine, University of Calgary, Calgary, Alberta, Canada; 4 Adult Cystic Fibrosis Clinic, University of Calgary, Calgary, Alberta, Canada; 5 Department of Biological Sciences, University of Calgary, Calgary, Alberta, Canada; 6 Department of Biochemistry and Molecular Biology, University of Calgary, Calgary, Alberta, Canada; Stanford University, United States of America

## Abstract

A number of human infections are characterized by the presence of more than one bacterial species and are defined as polymicrobial diseases. Methods for the analysis of the complex biological interactions in mixed infections with a large number of microorganisms are limited and do not effectively determine the contribution of each bacterial species to the pathogenesis of the polymicrobial community. We have developed a novel *Drosophila melanogaster* infection model to study microbe–microbe interactions and polymicrobe–host interactions. Using this infection model, we examined the interaction of 40 oropharyngeal isolates with *Pseudomonas aeruginosa*. We observe three classes of microorganisms, one of which acts synergistically with the principal pathogen, while being avirulent or even beneficial on its own. This synergy involves microbe–microbe interactions that result in the modulation of *P. aeruginosa* virulence factor gene expression within infected *Drosophila*. The host innate immune response to these natural-route polymicrobial infections is complex and characterized by additive, suppressive, and synergistic transcriptional activation of antimicrobial peptide genes. The polymicrobial infection model was used to differentiate the bacterial flora in cystic fibrosis (CF) sputum, revealing that a large proportion of the organisms in CF airways has the ability to influence the outcome of an infection when in combination with the principal CF pathogen *P. aeruginosa*.

## Author Summary

Bacterial infections often involve more than one species. The lung disease of cystic fibrosis (CF) patients provides examples of polymicrobial infections whereby diverse and dynamic microbial communities are a characteristic of CF airways. The significance of microbe–microbe interactions and the interplay of the communities with the host have not been thoroughly investigated. We describe a novel *Drosophila* model to discern the biological interactions between microbes within microbial communities, as well as the interactions between the communities and the innate immune system. Using fly survival as a readout of relevant interactions, we show that mixed infections may additively or synergistically enhance the pathogenicity of a microbial community. The polymicrobial infection model was used to differentiate the bacterial flora in CF sputum, revealing that a large proportion of the organisms in CF airways has the ability to influence the outcome of an infection when in combination with the principal CF pathogen *Pseudomonas aeruginosa*. We show that during the synergistic-type mixed infections, *P. aeruginosa* virulence gene expression is altered within live *Drosophila* compared to mono-species infections. The immune response to microbial communities takes many forms and can include synergistic activation of antimicrobial peptide gene expression. We postulate that the biological interactions exposed using this model may contribute to the transition from chronic stable infections to acute pulmonary exacerbation infections in CF.

## Introduction

Infections marked with more than one bacterial species are common. Suitable models are required to study the microbe–microbe interactions within these mixed infections, as well as the complex interplay between the polymicrobial communities and the host immune system [1]. Results from both molecular typing and microbiologic techniques on endobrochial secretions have defined cystic fibrosis (CF) lower airway disease with polymicrobial etiology [2]–[8]. In patients with CF, defective mucocilliary clearance [9] and impaired innate immunity [10], lead to chronic pulmonary infections. These are characterized by long periods of stability (despite high bacterial loads) that are punctuated by episodes of overt immunologic responses that cause the majority of irreversible lung damage. It is because of these repeated cycles that 90% of CF patients progress to pulmonary failure [11]. Aside from respiratory viruses, which may account for up to a third of exacerbations, the factors triggering the transition from a chronic stable infection to an acute pulmonary exacerbation remain elusive. Notwithstanding consistent detection at clinically significant levels [12] the role of the majority of bacterial species in the CF lung, mostly representatives of the oropharyngeal flora (OF), have not been defined. We previously showed that Viridans group streptococci and coagulase-negative staphylococci represent noteworthy classes of OF due to their capacity to modulate the gene expression of the principal pathogen *Pseudomonas aeruginosa*, which results in enhanced expression of many important virulence factors. Using a rat agar bead model of infection, we demonstrated that co-infection with an OF strain and *P. aeruginosa* caused a synergistic enhancement of lung inflammation [12]. The complexity of polymicrobial infections, such as those in CF, make them difficult to study and there are practical limits to the use of mammalian models for an adequate dissection of the multifarious biological interactions.

*P. aeruginosa* is responsible for most of the morbidity and mortality associated with CF lung disease; 80% of patients develop *P. aeruginosa* infections by early adulthood that persist for decades in spite of aggressive clinical interventions [13]. *P. aeruginosa* is capable of causing disease in plants [14], the nematode worm *Caenorhabditis elegans* [15], the amoeba *Dictyostelium discoideum* [16], and a number of insects [17]. Regardless of the diverse host range, *P. aeruginosa* utilizes common virulence mechanisms [16],[17] and genes necessary for mammalian pathogenesis are also essential for pathogenicity in the fruit fly [17],[18].

The evolution Gnathostomes (jawed vertebrates) is augmented by both adaptive and innate immune responses, whereas invertebrates solely depend on mechanisms of innate immunity. *Drosophila* mounts a complex multi-component response to bacterial infection, involving antimicrobial peptides (AMPs), hemocytes and phenoloxidase-based melanization [19]–[23]. The principles of *Drosophila* innate immunity exposed the central role of Toll-like receptors (TLRs) in humans for their ability to recognize non-self microbial antigens as pathogen associated molecular patterns (PAMPs) [24]. *Drosophila* can discriminate between various classes of microorganisms [25] which results in the transcriptional activation of AMP genes depending on the nature of the foreign invader [26]. The Toll pathway and the *immune deficiency* (IMD) pathways can act synergistically or separately to induce the expression of AMPs [27]. The precise transcriptional activation profile is largely in part due to a balance of inputs from the transcription factors Dorsal, DIF, and Relish [27]–[29]; Dorsal and DIF are regulated by the Toll pathway and Relish is activated by the IMD pathway [30]–[33]. The total output of immune activation by specific PAMPs seems to result from both pathogen recognition and pathology induced signaling [34].

The aim of this work was to develop a model system in which to discern biologically relevant microbe–microbe interactions, as well as investigate the interactions between microbial communities and the host. We have adapted a *Drosophila* natural-route infection model as a novel experimental system to examine these interactions during mixed infections and use the microbial communities in CF airways as an example of how such a model may help elucidate the clinical course of polymicrobial disease.

## Results/Discussion

### *Drosophila* natural-route *P. aeruginosa* infection

**Figure 1:**
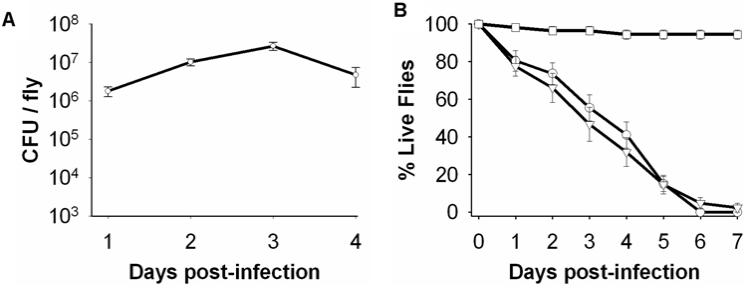
Characterization of the *P. aeruginosa Drosophila* infection. (A) The CFU/fly during the first four days of feeding on PA01. (B) Survival curves of *Drosophila* chronically infected with PA01: open boxes, flies fed 5% sucrose; open circles, flies continuously exposed to PA01; open triangles, flies exposed to PA01 for 24 hours and then transferred to sterile food.

*Drosophila* has been adopted as a model to identify *P. aeruginosa* mutants with reduced virulence and to analyze the interactions between this bacterium and innate host defenses. Feeding *P. aeruginosa* to *Drosophila* demonstrated the contribution of quorum sensing, the stringent response, and possibly pyocyanin to pathogenesis in the fly [35],[36]. We adapted the feeding assay, originally developed by Chugani *et al.* [37], to a 24-well plate format to accommodate screening large numbers of infections. We used wild-type *P. aeruginosa* strain PA01 for all infections described in this work. Initially, we characterized the amount of *P. aeruginosa* per fly during the first four days post-infection (Figure 1A). The CFU/fly rapidly reaches greater than 10^6^ CFU/fly 24 hours post-infection, and remains within 10^6^ to 10^7^ CFU/fly during the first 4 days. *Drosophila* is chronically infected by PA01; if flies are removed from the PA01 24 hours post-infection and transferred to wells containing sterile food, the survival curve mirrors that of flies that are constantly exposed to PA01 (Figure 1B) demonstrating that a chronic infection is established.

**Figure 2:**
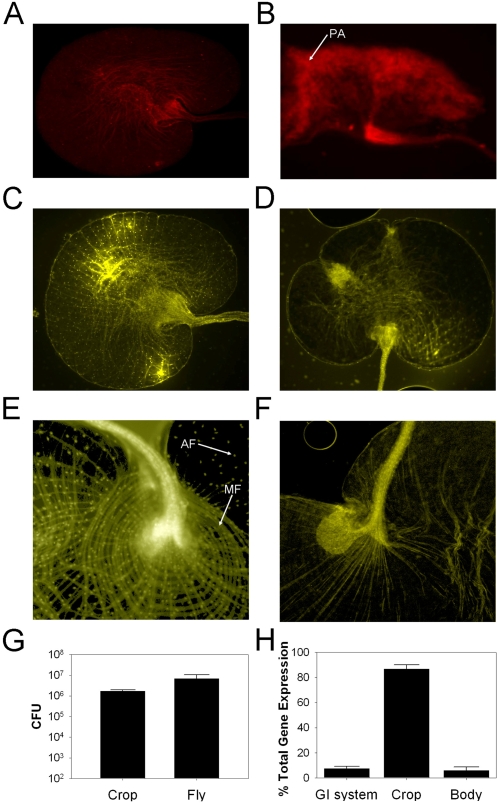
Fluorescence microscopy and direct measurement of *P. aeruginosa* infected *Drosophila*. (A) Red auto-fluorescence from an uninfected *Drosophila* crop (10×). (B) mCherry fluorescence from *P. aeruginosa* in a *Drosophila* infected crop (10×). (C,E) Yellow auto-fluorescence from an uninfected crop, 10× and 40× respectively. (D,F) Yellow auto-fluorescence from an infected crop, 10× and 40× respectively. Crops were harvested from twenty infected flies 24 hours post-infection and bacterial load was measured. The PAO1 in the crops and in whole flies were determined by plating (G). The entire gut (the foregut, the midgut and the hindgut), the crop, and remaining fly body were removed from twenty flies infected with a PA01 strain expressing luciferase from the *lasI* promoter (24 hours post-infection) and gene expression measured for each individually (H).

Natural-route infection of the *Drosophila* digestive system can result in morphological alterations including loss or degeneration of epithelial cells and loss of typical intestinal shape [38]. Few microbes have been identified that naturally infect *Drosophila*, however, oral infection with *Pseudomonas entomophila* (pathogenic to *Drosophila* larvae) causes a cessation in food-uptake [39]. Epithelial cells are absent or display abnormal microvilli as compared to uninfected larvae [40]. With the exceptions of wasting during *Mycobacterium marinum* infection [41], the presumed fluid loss during *Vibrio cholera* infection [42] and the digestive abnormalities resulting from *P. entomophila* infections, little else is known about the cause of death of infected *Drosophila* [34]. The pathophysiology resulting from *P. aeruginosa* natural-route infection has not been investigated. To this end, *Drosophila* was infected with a *P. aeruginosa* strain expressing the mCherry fluorescent protein. Fluorescence microscopy revealed that the predominant site of infection was the crop (Figure 2A and 2B), a food storage organ. The crop is lined with an epithelium; we serendipitously observed that the cell nuclei comprising this layer are clearly evident as yellow auto-fluorescent foci in uninfected crops (Figure 2C and 2E). The epithelial layer is destroyed or severely damaged upon infection, as seen by the absence of epithelial cell nuclei in *P. aeruginosa* infected crops (Figure 2D and 2F). The musculature structure in the crop is composed of wide bands of circular muscles that cover the wall of the crop with a plexus of branched and interlacing fibers (Figure 2E); this architecture is absent in infected crops (Figure 2F). Systemic infection by microbial invasion into the hemocoel through crossing the gut epithelium is not required to kill flies in other situations [38]. Therefore, the destruction of the epithelial layer and the musculature of the crop likely impairs normal digestive function leading to death but a role for systemic infection cannot be ruled out.

To further confirm that the crop was the predominant site of *P. aeruginosa* colonization we removed crops from twenty infected flies 24 hours post-infection and measured the PA01 bacterial load. We observed that the crop was colonized by >10^6^ CFU, similar to the total bacterial load detected in whole animals (Figure 2G). Additionally, the entire gut (the foregut, the midgut and the hindgut) was removed from twenty flies infected with a PA01 strain expressing luciferase from the *lasI* promoter (24 hours post-infection). The crop was removed from the gastrointestinal system (GI) leaving the proventriculus attached to the intact GI system. The crops, the remaining GI systems and the fly carcasses were individually transferred to the wells of a 96-well plate and luciferase activity was measured. This analysis revealed that the majority of the detectable *P. aeruginosa* gene expression (>86%) is localized to the crop (Figure 2H).

### *Drosophila* as a surrogate host for polymicrobial infections

Polymicrobial infection can involve complex interactions between microbes as well as between the microbes and the host. We previously implicated members of the OF population for contributing to polymicrobial infections through their ability to enhance *P. aeruginosa* virulence [12]. To further systematically investigate the OF population in CF we cultivated OF strains from sputum samples collected from patients chronically colonized with *P. aeruginosa*. Organisms at concentrations greater than 10^6^ CFU/ml of sputum were purified and identified through sequence of a partial fragment of the universal bacterial 16S rRNA gene. Many of these organisms could only be classified to the genus level. Cultivation conditions and the GenBank accession numbers for each isolate are provided in Table S1.

**Figure 3:**
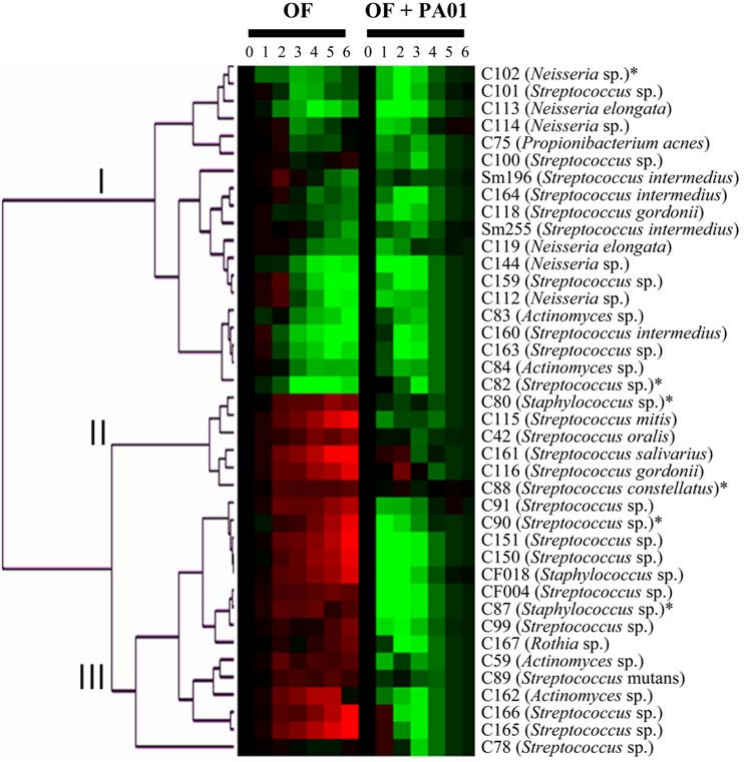
Cluster representation of the three OF infection classes. The survival curve of each OF infection alone was compared to the survival curve of flies feeding on 5% sucrose. The survival curves of the OF PA01 co-infections were compared to the survival curve of PA01 alone. Green boxes indicate time points where fewer flies were alive as compared to controls; red indicates time points where more flies were alive as compared to controls. The data was collected from six independent infections with a minimum of 25 flies per infection. The * indicate infections that were further characterized.

Forty OF isolates were fed to *Drosophila* alone as well as in combination with PA01 and survival was monitored daily. The resulting survival curves were compared to the curves for flies fed only 5% sucrose and PA01 alone. The relative differences between the infections and the controls are presented in Figure 3. OF strains clearly belong to one of three infection classes. Class I (virulent) represent OF organisms that alone are able to kill flies and flies are killed faster in the presence of PA01 as compared to PA01 alone. Class II (avirulent) represent OF strains that alone are unable to kill flies and in combination with PA01 do not enhance killing. Class III (synergistic) represent organisms that alone are not pathogenic to *Drosophila* but in combination with PA01 dramatically reduce fly survival.

We included strain CF004 in our co-infection assay. We have previously demonstrated that this strain enhances *P. aeruginosa* virulence factor gene expression during co-culture [12]. In an agar bead infection model in rat lungs, CF004 alone caused little to no lung consolidation. However, in combination with *P. aeruginosa* a dramatic increase in tissue destruction was detected, an effect that could not be explained by the additive effect of CF004 and PA01 pathogenicity [12]. CF004 behaves in the fly model similar to the rat lung model and belongs to class III, thus the synergistic polymicrobial infection seen in mammalian lungs can be established in *Drosophila*. The fly feeding assay represents a high throughput reproducible infection model for studying polymicrobial infections. Using *Drosophila* as a surrogate host for polymicrobial infections revealed that 38% of the OF strains tested have the capacity to act synergistically with *P. aeruginosa* and 48% of OF strains are potentially pathogenic, although none were as virulent as *P. aeruginosa* (Figure S1).

**Figure 4:**
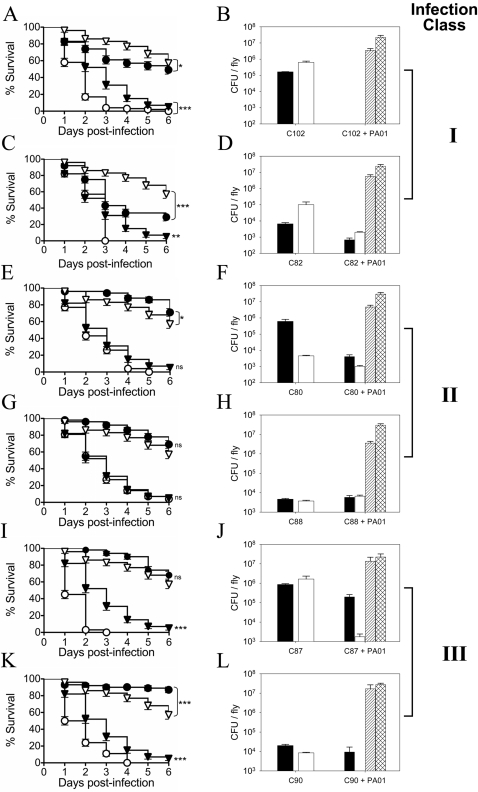
Kaplan-Meier survival curves and quantitative bacteriology of two representative OF strains for each infection class. Open triangles, survival curve of flies feeding on 5% sucrose; filled triangles, survival of flies infected with PA01; filled circles, survival of flies infected with the OF strain alone; open circles, survival of flies co-infected with PA01 and the OF strain. The black bar shows the OF CFU/fly 24 post-infection; black bars with white hatches, OF CFU/fly 48 post-infection; open white bars, PA01 CFU/fly 24 hours post-infection; white bars with black hatches, PA01 CFU/fly 48 hours post-infection. The C102 bacterial load was unable to determined in monoinfections due to PA01 overgrowth. (A–D) are Class I organisms; (E–H) are Class II organism; (I–L) are Class III organisms. Log-rank analysis (Mantel-Cox) was used to compare OF infections with the sucrose control and mixed infections with the PA01 control; statistical significance between survival curves is shown with * P<0.05, ** P<0.005 and *** P<0.0005; ns = not significant.

Two representative OF strains from each class were further characterized. Infected flies were homogenized and planted on selective media for enumeration of the OF strain and PA01 at 24 and 48 hours post-infection. The survival curves of C102 (*Neisseria* sp.) and C82 (*Streptococcus* sp.) (Figure 4A and 4C) belong to class I and kill flies in the absence of PA01. Both OF strains numerically increase during the first 48 hours of the infection (Figure 4B and 4D), suggesting an ability to effectively colonize *Drosophila*. C82 numbers are reduced 100 fold at both the 24 and 48 hour time points in the presence of PA01 (Figure 4D). Strains C80 (*Streptococcus* sp.) and C88 (*Streptococcus constellatus*) belong to class II and are unable to alter the survival curves as compared to controls (Figure 4E and 4G). In the case of C80, either the fly immune response and or the inability to effectively colonize cause a reduction in OF numbers during the infection (Figure 4F). C88 appears to poorly colonize *Drosophila* (Figure 4H); both C80 and C88 are detected in low abundance in the presence of PA01, as might be expected for organisms that do not significantly alter the survival curves (Figure 4F and 4H). C87 (*Staphylococcus* sp.) and C90 (*Streptococcus* sp.) belong to class III. The most interesting group of OF strains because they do not appear to be pathogenic to *Drosophila*, however, in mixed infections with PA01 they significantly enhance fly killing (Figure 4I and 4K). In fact, C90 on its own appears to be beneficial resulting in increased fly survival compared to the control. C87 and C90 are both detected in *Drosophila* during infections with only the OF strain (Figure 4J and 4L); notably C87 is present at concentrations greater than 10^6^ CFU/fly 48 hours post-infection without detectable fly mortality. Interestingly, in co-infections both organisms show a greatly reduced prevalence 48 hours post-infection (10,000-fold); C90 is not detectable within 48 hours (Figure 4J and 4L). This may be due to the immune response mounted by *Drosophila* in these mixed infections or alternatively, the OF stains are being killed by PA01 within the fly. PA01 numerically behaves in all other mixed infections as PA01 does alone (Figure 1A) and the *P. aeruginosa* bacterial load in co-infected crops does not significantly change in the presence of any of the OF strains tested (data not shown).

The enhanced fly killing seen with Class I organisms is likely due to an additive effect of the OF and PA01 pathogenicity. The enhanced killing seen with Class III organisms cannot be simply explained. A number of possibilities could contribute to this effect. First, PA01 gene expression may be altered as a consequence of the polymicrobial environment, which may enhance virulence, a phenomenon that occurs with two members of this class, CF004 and CF018 [12]. Alternatively, the reverse is also possible whereby the normally avirulent OF species becomes pathogenic in response to PA01. Second, the immune response to combinations of organisms may lead to hyperactivity and produce a detrimental effect to the fly. In humans, much of the damage resulting from bacterial infection is often due to the response of the immune system rather than a direct action of the pathogen. It is very interesting that some Class III organisms have a beneficial effect on *Drosophila* survival when independent of PA01. This is consistent with the observation that exposure of wild-type flies (Canton S) to bacteria during the first week of adult life (the flies infected in this study were in the first week of life) can enhance longevity by 30–35% in contrast to flies reared under axenic conditions [43]. It is unclear if the Class III organisms are beneficial due to an added nutritional value or if their beneficial effect is the result of stimulating advantageous signaling pathways in the fly.

### *In vivo* modulation of *P. aeruginosa* virulence factor gene expression during synergistic-type infections

We initially chose to address if the reduced fly survival in PA01-Class III (synergistic) mixed infections may result from altered *P. aeruginosa* gene expression. To this end, the gene expression of 24 *P. aeruginosa* virulence factors was measured in real-time, within live flies, during infections with PA01 alone or in combination with Class III organisms (C90 and C87, Figure 4). *P. aeruginosa* virulence factor reporter strains (Table S2) were constructed as single copy chromosomal *luxCDABE* fusions using the CTX integration system [44],[45]. Groups of flies were infected with *P. aeruginosa* reporter strains for 24 hours before individual live flies were transferred to the wells of 96-well plates containing filters soaked in either 5% sucrose, or a bacterial suspension of either C90 or C87. Luminescence was measured hourly post-transfer to the 96-well plates. This infection strategy was used for several reasons. First, we know that a 24 hour exposure to PA01 is sufficient to establish a chronic infection in *Drosophila* (Figure 1B) and removing flies from the *P. aeruginosa* (on the filters) ensures that the measured luminescence does not originate from the filter but rather from *P. aeruginosa* within flies. The amount of detectable luciferase on filters in the 96-well plates was measured 30 hours post-transfer following the removal of all flies. This represented 0.96%+/−0.49 of the total luciferase activity before the removal of flies, suggesting that *P. aeruginosa* gene expression on the filters (which do not support *P. aeruginosa* growth) is not a significant contribution in the assay. Second, we chose to measure the luminescence from individual flies and not groups of flies because real-time measurements in single animals permitted us to integrate the viability of each fly into the data. Therefore, by scoring when each animal died the data could be used to represent *P. aeruginosa* gene expression in only live flies. The experimental design also allowed us to use the data to represent *P. aeruginosa* virulence factor gene expression just prior to death—not possible if we were to harvest organisms in order to measure gene expression (for example to isolate mRNA). Pooled measurements or harvesting would have merely given a perspective on *P. aeruginosa* global gene expression at a single time point in a population of flies, all at different stages of infection.

**Figure 5:**
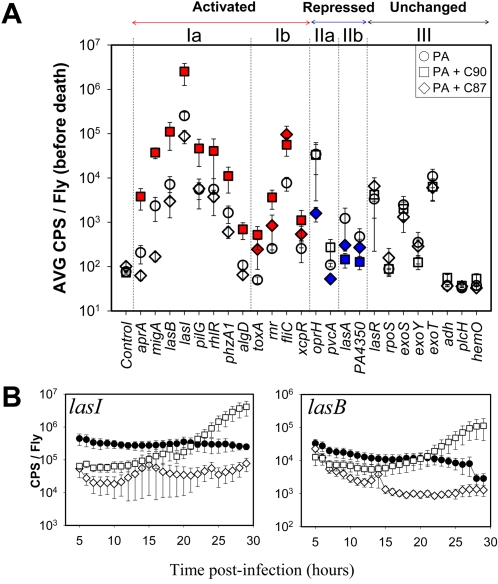
*In vivo* modulation of *P. aeruginosa* virulence factor gene expression by OF. (A) The level of gene expression of 24 *P. aeruginosa* virulence factors in *Drosophila* prior to death during infection with *P. aeruginosa* alone (circles) or co-infection with C90 (squares) or C87 (diamonds). The average CPS/Fly before death was calculated from the average CPS/Fly during the last two hours of life from eight flies per condition. Red and blue symbols indicate promoters activated or repressed in the presence of the OF respectively. (B) Temporal expression of *lasI* and *lasB* in *Drosophila* infected with *P. aeruginosa* alone (black circles) and in the presence of C90 (open squares) or C87 (open diamonds). The * indicate those profiles considered to be statistically significant (Student's t test, p<0.05) compared to infections with *P. aeruginosa* alone.

The exquisite sensitivity of using luciferase as a reporter is highlighted by the ability to measure the expression of the 24 virulence factors through greater than five orders of magnitude (Figure 5A). The *P. aeruginosa* virulence factors can be assigned to one of four classes based on their expression profile during mixed infections just prior to fly death (Figure 5A). Class Ia are genes activated only in the presence of C90 (*Streptococcus* sp.) above the levels in flies infected with *P. aeruginosa* alone. Interestingly, a number of quorum sensing regulated genes such as *lasI*, *lasB*, *rhlR* and *phzA1* belong to this class and suggest that quorum sensing circuits in C90 PA01 mixed infections are upregulated. Figure 5B shows the expression profile of *lasI* (acyl-homoserine lactone synthase) and *lasB* (elastase), two quorum sensing regulated genes, during the first 30 hours post-exposure to the OF strains. Clearly, both genes are only activated in the presence of C90 after approximately 20 hours. In a previous study, we implicated some of these quorum sensing regulated genes (*phzA1* and *lasB*) to be responsive to the universal bacterial signal AI-2 (which is produced by both C90 and C87 and not *P. aeruginosa*) [12]. It will be interesting to investigate if AI-2 is in part responsible for the modulation of these promoters in *Drosophila*.

Class Ib (Figure 5A) are genes activated in the presence of either OF organisms (C90 or C87). The most significant of these appears to be *fliC* (encodes a flagellar filament protein), suggesting that motility might be upregulated just prior to death in both OF PA01 mixed infections. We also observed two small classes of genes that were repressed in the presence of only C87 (Class IIa) or both C90 and C87 (Class IIb) revealing that it may not only be activation of *P. aeruginosa* virulence factors that contribute to the enhanced pathogenicity of synergistic-type mixed infections. Finally, we were unable to detect any change in Class III genes (Figure 5A) during mixed infections. The *exoS*, *exoY* and *exoT* genes, encoding exoenzymes, belong to this class. The *exoS* gene expression profiles are provided as a representative of the unresponsive promoter class (Figure S2). Although these genes do not seem to be activated during mixed infections, it is important to consider that their contribution to fly killing might still be significant but may occur earlier in OF PA01 co-infected flies.

We have shown that *P. aeruginosa* virulence factor gene expression is altered upon exposure to OF organisms *in vivo*. C90 and C87, both organisms with the capacity to synergistically enhance fly killing in the presence of *P. aeruginosa*, show very different capacities to modulate PA01 gene expression. It is therefore unlikely that a single mechanism is responsible for the decreased survival seen in Class III PA01 co-infections.

### Innate immune response to polymicrobial infection

**Figure 6:**
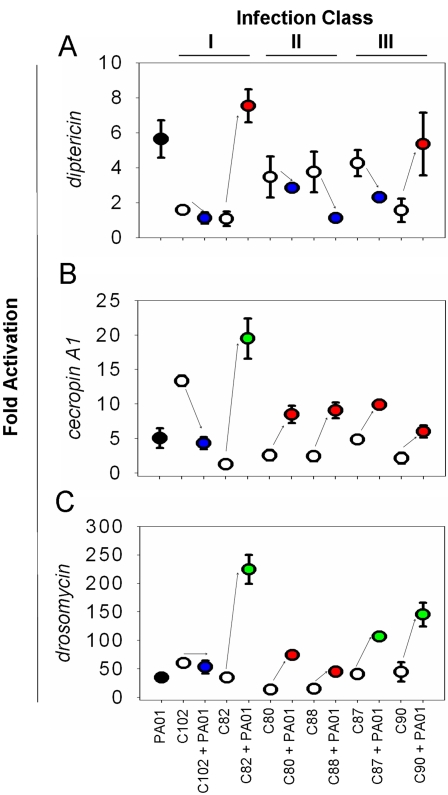
*Drosophila* AMP gene expression 24 hours post-infection. Cecropins have a broad spectrum of activity against Gram-positive, Gram-negative, protozoan parasites, and fungi and are induced in the intestine during gut infections [38]. Real-time PCR was used to calculate the fold transcriptional activation above uninfected flies: (A) *ditericin* expression; (B) *cecropin A1* expression; (C) *drosomycin* expression. Solid black circles indicate the response to PA01 alone; blue circles, examples of suppressed AMP expression during co-infection; red circles, AMP expression that can be explained by an additive effect; green circles, synergistic activation of AMP expression.

*Drosophila* is capable of expressing seven distinct classes of AMPs in response to microbial infection that include: Cecropins [46], Diptericin [47], Drosocin [48], Defensin [49], Metchnikowin [50], Attacin [51], and Drosomycin [52]. We exploited the utility of *Drosophila* AMP gene expression to address how a model innate system responds to polymicrobial infections and how such a response might contribute to the altered survival during mixed infection. Total RNA was extracted 24 hours post-infection from the quantitative bacteriology experiment (Figure 4) and we performed TaqMan Real-time PCR using probes for *diptericin*, *cecropin A1*, and *drosomycin*. Transcriptional activation is represented as a fold change relative to the constitutive levels of AMP gene expression in uninfected flies (Figure 6).

*P. aeruginosa* stimulates the transcriptional activation of all AMPs tested; *diptericin* is induced 4.56–6.7 fold (Figure 6A), *cecropin A1* is induce 3.6–6.45 fold (Figure 6B), and *drosomycin* is activated 30.03–39.16 fold (Figure 6C). Unexpectedly, we observed three different types of immune responses to polymicrobial infections: increased AMP expression during mixed infections that could be explained by an additive effect of the response to PA01 and the OF strain; a suppression of the AMP activation (a mechanism used by commensal microbes in the *Drosophila* gut [53]); and synergistic activation of the AMP expression. For *diptericin* expression, all but the C82 and C90 mixed infections have reduced transcriptional activation in the polymicrobial infections (Figure 6A). Interestingly, PA14, a highly virulent strain of *P. aeruginosa*, seemingly has the capacity to suppress AMP expression in *Drosophila* during systemic infection [54]. A similar phenotype is observed using PA01 in the feeding assay only if OF strains are present (Figure 6A). Expression of *cecropin A1* shows the additive induction response for most infections with the exception of the C102 and C82 mixed infections. Recall that C102 is a *Neisseria* sp. and as expected induces a potent *cecropin* response on its own (12.57–14.07 fold). However, when in combination with PA01 we see a suppression response; *cecropin* is expressed at levels comparable to the infection with PA01 alone (Figure 6B). We also note an example of synergistic *cecropin* activation in the C82 mixed infection; C82 alone does not induce *cecropin* (1.05–1.44 fold), however in combination with PA01 *cecropin* is induced 16.57–22.36 fold. This represents a 3 fold increased induction of *cecropin* as compared to the PA01 infection. All OF strains tested induce a strong *drosomycin* response and for the most part, the induction in the mixed infections can be explained as an additive effect (Figure 6C). However, in three of the polymicrobial infections (C82, C87, and C90), *drosomycin* expression shows synergistic activation. Interestingly, both infections with organisms (C87 and C90) belonging to OF class III (not pathogenic to flies alone but enhance pathogenicity with PA01) and C82 (the most aggressive mixed infection aside from the C87 mixed infection (Figure 4I)) when associated with PA01 activate *drosomycin* transcription to levels that are not approached by adding the OF and PA01 *drosomycin* activations. It has been suggested that the immune response mounted by *Drosophila*, in some cases, may be detrimental and the ultimate cause of death [53],[55]; our data seems to support this hypothesis. It is interesting that we observed such strong activation of *drosomycin*, an antifungal agent; the function of Drosomycin in the polymicrobial infections is unclear. Synergistic activity of AMPs have been reported [56]. Perhaps Drosomycin has alternative activity in the milieu of other AMPs. Alternatively, Drosomycin may serve secondary functions in the immune system, as AMPs are known to do in vertebrates [57]. Supporting the value of the *Drosophila* as a surrogate host for polymicrobial infections, synergistic activation of innate immune responses have been reported during polymicrobial colonization of human epithelial cells [58]. The response of the mammalian innate immune system to combinations of TLR ligands (and or other pattern recognition ligands) can cause both synergistic activation and synergistic down regulation of a number of genes involved in regulating both innate and adaptive immune reactions to pathogenic microbes. The molecular mechanisms mediating such outcomes are not well understood but likely involve the cooperation of multiple signal transduction pathways [59]. Recently, it has been shown that co-stimulation of the Toll and IMD pathways results in synergistic transcriptional activation of AMPs such as Drosomycin, Diptericin, and Cecropin mediated through the mechanism of utilizing the NF-κB transcription factors DIF, Dorsal, and Relish. Synergistic AMP expression does not occur during septic injury infections; exposure to *Beauveria bassiana* spores and feeding on *P. entomophila* was required to detect the response in live animals [27]. The development of a *Drosophila* mixed infection model provides a means to further explore the phenomenon of synergy in innate immunity.

### Concluding remarks

Commensal bacterial flora have long been recognized for their immune stimulatory role and as barriers to invading pathogens. In CF, the OF colonizes normally sterile parts of the airway and should be considered more carefully [2]–[6],[8],[12]. The complex microbial communities in the CF lung provide an environment whereby the principal pathogen, *P. aeruginosa*, can interact with a number of bacterial species. The inability to detect a pathogenic response of these organisms in conventional infection models should not disregard their potential to contribute to the polymicrobial infection. Understanding the complex interplay between pathogen(s), normal flora, and the immune system may be crucial for improving the therapy of polymicrobial diseases such as CF.

We have shown that a number of OF isolates that are avirulent or beneficial to the fly have the capacity to synergistically enhance the pathogenicity of a microbial community. By further characterizing two examples of these synergistic-type organisms it is clear that the mechanisms at play to explain this synergy can be very different in terms of microbe–microbe interactions and polymicrobe–host interactions. Both C90 and C87 produce almost identical phenotypes in the natural-route *Drosophila* infection model as measured by host survival. However from the perspective of the co-infecting pathogen *P. aeruginosa* (as measured by virulence factor gene expression) or from the perspective of the host's innate immune system (as measured by antimicrobial peptide response) they are very different infections. This result highlights the potential complexity of polymicrobial infections. The *Drosophila* model of polymicrobial infections not only allows relevant microbe–microbe interaction to be easily discerned based on fly survival but also provides a framework to further discriminate these interactions by assaying both bacterial and host gene expression *in vivo*.

## Materials and Methods

### Bacterial strains and culture conditions

CF sputum samples were collected in sterile containers, sheared with vigorous passage through a 1cc syringe (without a needle), serially diluted, and cultured on several standard media types: MacConkey Agar (Becton Dickinson and Company (BD)), Pseudomonas Isolation Agar (PIA) (BD), Mannitol Salt Agar (BD), Chocolate Agar (BD), Columbia 5% Blood Agar (BD), Brain Heart Infusion Agar (BHI) (BD), and Trypticase Soy Yeast Agar (Trypticase Soy Agar (BD), 3 g/L yeast extract (TSY)). Plates were incubated at 37 °C with 5% CO_2_ for at least 5 days. Single colonies were purified three times on the selective medium they were isolated on. Broth cultures were grown in the media shown in Table S1. Bacterial strains were identified by PCR amplification of a part of the 16S rRNA gene using primers 8f and 926r [60]. BlastN (http://www.ncbi.nlm.nih.gov/BLAST/) was used compared the sequence of the PCR product to publicly available sequences. PA01 [61] was used as the wild-type *P. aeruginosa* strain for all the experiments; PA01 was grown in BHI at 37 °C. *Pac*I fragments encompassing the virulence factor promoter fused to the *luxCDABE* operon in pMS402 [12] were ligated to a 6255 bp *EcoR*V (*Pac*I linker added) from mini-CTX*lux* [44] which encodes the origin of replication, tetracycline (Tc) resistance, and the CTX integrase. For fluorescence microscopy the *Not*I *luxCDABE* cassette (under control of to the *pilG* promoter) was replaced with the mCherry ORF via engineered NotI restriction sites [62]. The recombinant plasmid was transferred from an *Escherichia coli* SM10 donor to PA01 via a biparental mating as previously described [45]. Transconjugants were selected for on PIA containing Tc (200 µg/ml).

### Fly infections

Infections were adapted from the fly feeding assay developed by Chugani et al [37]. Broth cultures (both OF and PA01) were adjusted to an OD_600_ = 2.0 using the media the strain was grown in. For infections with a single strain, 1.5 ml of the culture was collected by centrifugation, the supernatant removed and the pellet was resuspended in 100 µl 5% sucrose. For co-infections, 1.5 ml of adjusted PA01 culture was collected, the supernatant was removed and 1.5 ml of the adjusted OF culture was added to the tube containing the PA01 pellet and the OF strain was pelleted by centrifugation. The resulting pellet was resuspended in 100 µl of 5% sucrose. The resuspended cells were spotted onto a sterile filter (Whatman GF/A 21 mm) that was placed on the surface of 1.5 ml of solidified 5% sucrose agar in the well of a 24-well plate (Falcon Cat No. 351147). The plates were placed at 37 °C for 30 minutes. Male Canton S flies (3–5 days old) were starved for 3 hours before 10–14 flies were added to each well of the 24-well plate. Carbon dioxide was used for anesthetizing flies throughout the sorting and transferring process. Infection plates were stored at 26 °C in a humidity controlled environment. The number of live flies to start the experiment was documented and live flies were counted at 24 hour intervals. For each infection group a minimum of 6 wells (greater than 60 flies) were used. For the *in vivo P. aeruginosa* virulence gene expression experiments groups of 50 male flies were initially infected in vials (VWR Cat No. 16004-036) for 24 hours with each reporter strain as described above. Single flies was transferred to wells of black NUNC 96-well plates (with lids) containing 100 µl of 5% sucrose agar per well overlaid with a filter paper. The filter was spotted with 20 µl of either 5% sucrose or a 5% sucrose suspension of C90 or C87 (OD600 = 2.0) and allowed time to air dry. A minimum of eight flies were used for each test condition. Following transfer to the 96-well plate viability was scored and luminescence was measured once an hour with a 1450 Microbeta Trilux Liquid Scintillation and Luminescence Counter (Wallac). The data is a representative of at least two independent experiments.

### Cluster analysis

Cluster analysis was used to assign OF organisms to an infection class using Cluster [63] and Treeview [63]. Controls (sucrose or PA01 alone) were used as a base line; the numbers used in clustering are the results of test minus controls (OF alone compared to sucrose; PA01+OF compared to PA01). Uncentered complete linkage hierarchical clustering analysis was performed.

### Fluorescence microscopy

The gastrointestinal (GI) system was dissected out of flies that had been exposed to PA01 expressing mCherry from the *pilG* promoter 48 hours post-infection. GI systems were placed under a cover slip and visualized with a Leica DM RXA2 microscope. Chroma Epi-illumination filter cubes 41028 and 41043 were used to visualize yellow and red fluorescence respectively. Crops were photographed under 10× dry and 40× NA PL FLUOTAR oil objectives.

### Quantitative bacteriology

Five infected live flies for each infection at 24 and 48 hours post-infection were crushed in 200 µl BHI, and serially diluted in BHI. Dilutions were spread onto PIA for PA01 enumeration, and BHI with colistin (1 µg/ml) and oxolinic Acid (0.5 µg/ml) for OF enumeration. PIA plates were incubated at 37 °C for 24 hours. BHI plates were incubated for 3 days at 37 °C in the presence of 5% CO_2_. Colonies were counted following incubation and CFU/fly was calculated.

### Real-time PCR

Total RNA was extracted from five flies for each infection 24 hours post-infection using TRIzol (Invitrogen) as previously described [39]. cDNA was synthesized with a High Capacity cDNA synthesis kit (ABI Biosystems). 100 ng of cDNA was used as template in the Real-time PCR reactions. Custom TaqMan probes for *diptericin* (Dm01841768_s1), *cecropin A1* (Dm02609400_s1) and *drosomycin* (Dm01822006_s1) were used as recommended by the manufacturer (ABI Biosystems). RpL32 (Dm02151827_g1) was used as the constitutive control; each reaction was done in triplicate and standard deviations were used to calculate a range of fold activation using the 2^ΔΔCt^ method [64].

### Statistical analysis

The results are given as mean±standard error of the mean. Student's t test analysis was performed and differences were considered significant when p<0.05. Survival data were analyzed using Kaplan-Meier survival curves with GraphPad Prism 5.0 (GraphPad Software Inc.) and significance was tested by Log-rank (Mantel-Cox) analysis.

## Supporting Information

Table S1 Bacterial strains used in this study.
(0.06 MB DOC)

Table S2 *P. aeruginosa* virulence factors examined in this study.
(0.04 MB DOC)

Figure S1 Killing curves for single organism infections of *Drosophila*.
(0.38 MB PDF)

Figure S2 An example of a *P. aeruginosa* promoter unaffected in OF co-infections.
(0.12 MB PDF)
