# Supplementary material for: Discerning the Complexity of Community Interactions Using a Drosophila Model of Polymicrobial Infections
Source: PLoS Pathog. 2008 Oct 24;4(10):e1000184. doi: 10.1371/journal.ppat.1000184 (PMC2566602; doi:10.1371/journal.ppat.1000184)
Supplement: Table S1 — Bacterial strains used in this study. (0.06 MB DOC) [file ppat.1000184.s001.doc]

**Table S1. Bacterial strains used in this study.**

| ***Strain Name*** | ***Media*** | ***GenBank Accession No.*** |
| --- | --- | --- |
| C102 (*Neisseria* sp.) | TSY | EF473978 |
| C101 (*Streptococcus* sp.) | TSY | EF473979 |
| C113 (*Neisseria elongata*) | BHI | EF473980 |
| C114 (*Neisseria* sp.) | BHI | EF473981 |
| C75 (*Propionibacterium acnes*) | BHI | EF473982 |
| C100 (*Streptococcus* sp.) | TSY | EF473983 |
| Sm196 (*Streptococcus intermedius*) | TSY | EF473984 |
| C164 (*Streptococcus intermedius*) | TSY | EF473985 |
| C118 (*Streptococcus gordonii*) | BHI | EF473986 |
| Sm255 (*Streptococcus intermedius*) | TSY | EF473987 |
| C119 (*Neisseria elongata*) | BHI | EF473988 |
| C144 (*Neisseria* sp.) | TSY | EF473989 |
| C159 (*Streptococcus* sp.) | TSY | EF473990 |
| C112 (*Neisseria* sp.) | BHI | EF473991 |
| C83 (*Actinomyces* sp.) | Blood | EF473992 |
| C160 (*Streptococcus intermedius*) | TSY | EF473993 |
| C163 (*Streptococcus* sp.) | TSY | EF473994 |
| C84 (*Actinomyces* sp.) | Blood | EF473995 |
| C82 (*Streptococcus* sp.) | Blood | EF473996 |
| C80 (*Staphylococcus* sp.) | Blood | EF473997 |
| C115 (*Streptococcus mitis*) | BHI | EF473998 |
| C42 (*Streptococcus oralis*) | Blood | EF473999 |
| C161 (*Streptococcus salivarius*) | TSY | EF474000 |
| C116 (*Streptococcus gordonii*) | BHI | EF474001 |
| C88 (*Streptococcus constellatus*) | TSY | EF474002 |
| C91 (*Streptococcus* sp.) | BHI | EF474003 |
| C90 (*Streptococcus* sp.) | BHI | EF474004 |
| C151 (*Streptococcus* sp.) | TSY | EF474005 |
| C150 (*Streptococcus* sp.) | TSY | EF474006 |
| CF018 (*Staphylococcus* sp.) | BHI | [12] |
| CF004 (*Streptococcus* sp.) | BHI | [12] |
| C87 (*Staphylococcus* sp.) | Blood | EF474007 |
| C99 (*Streptococcus* sp.) | TSY | EF474008 |
| C167 (*Rothia* sp.) | BHI | EF474009 |
| C59 (*Actinomyces* sp.) | Blood | EF474010 |
| C89 (*Streptococcus mutans*) | TSY | EF474011 |
| C162 (*Actinomyces* sp.) | TSY | EF474012 |
| C166 (*Streptococcus* sp.) | BHI | EF474013 |
| C165 (*Streptococcus* sp.) | BHI | EF474014 |
| C78 (*Streptococcus* sp.) | Blood | EF474015 |

^1^ Identification based on 16S rRNA gene sequence amplified with 8f and 926r primers[60]. Identification to the species level corresponds to >97% identity to the closest match in the RDP database (http://rdp.cme.msu.edu/).

^2^ Culture medium on which the strain was originally isolated from CF sputum
