# Supplementary material for: Discerning the Complexity of Community Interactions Using a Drosophila Model of Polymicrobial Infections
Source: PLoS Pathog. 2008 Oct 24;4(10):e1000184. doi: 10.1371/journal.ppat.1000184 (PMC2566602; doi:10.1371/journal.ppat.1000184)
Supplement: Table S2 — P. aeruginosa virulence factors examined in this study. (0.04 MB DOC) [file ppat.1000184.s002.doc]

**Table S2. *P. aeruginosa* virulence factors examined in this study.**

| ***Gene*** | ***Functional Description*** |
| --- | --- |
| *aprA* | alkaline protease (alkaline metalloproteinase precursor) |
| *migA* | probable glycosyl transferase (mucin-inducible gene) |
| *lasB* | elastase |
| *lasI* | AHL synthase |
| *pilG* | Type IV fimbrial (part of the *pilGHIJKL* gene cluster) |
| *rhlR* | AHL dependent transcriptional regulator |
| *phzA1* | pyocyanin biosynthesis |
| *algD* | alginate (GDP-mannose 6-dehydrogenase AlgD), first of 18-kb alginate operon. |
| *toxA* | exotoxinA |
| *rnr* | exoribonuclease RNase R |
| *fliC* | flagellar filament protein |
| *xcpR* | xcp (general secretion pathway protein E ) |
| *oprH* | PhoP/Q and low Mg2+ inducible outer membrane protein H1 precursor |
| *pvcA* | pyoverdine biosynthesis |
| *lasA* | protease |
| *PA4350* | transcriptional regulator |
| *lasR* | AHL dependent transcriptional regulator |
| *rpoS* | stationary phase sigma factor |
| *exoS* | exoenzymeS (ADP-ribosyltransferase) |
| *exoY* | adenylate cyclase, belongs to the exoenzyme S regulon |
| *exoT* | exoenzymeT (99% similar to ADP-ribosyltransferase (exoenzyme 53)) |
| *adh* | probable adhesion protein |
| *plcH* | hemolytic phopholipaseC (hemolysin) precursor |
| *hemO* | putative hemagglutinins (43% identity to *B. pertussis*) |
