# Supplementary material for: Discerning the Complexity of Community Interactions Using a Drosophila Model of Polymicrobial Infections
Source: PLoS Pathog. 2008 Oct 24;4(10):e1000184. doi: 10.1371/journal.ppat.1000184 (PMC2566602; doi:10.1371/journal.ppat.1000184)
Supplement: Figure S1 — Killing curves for single organism infections of Drosophila. (0.38 MB PDF) [file ppat.1000184.s003.pdf]

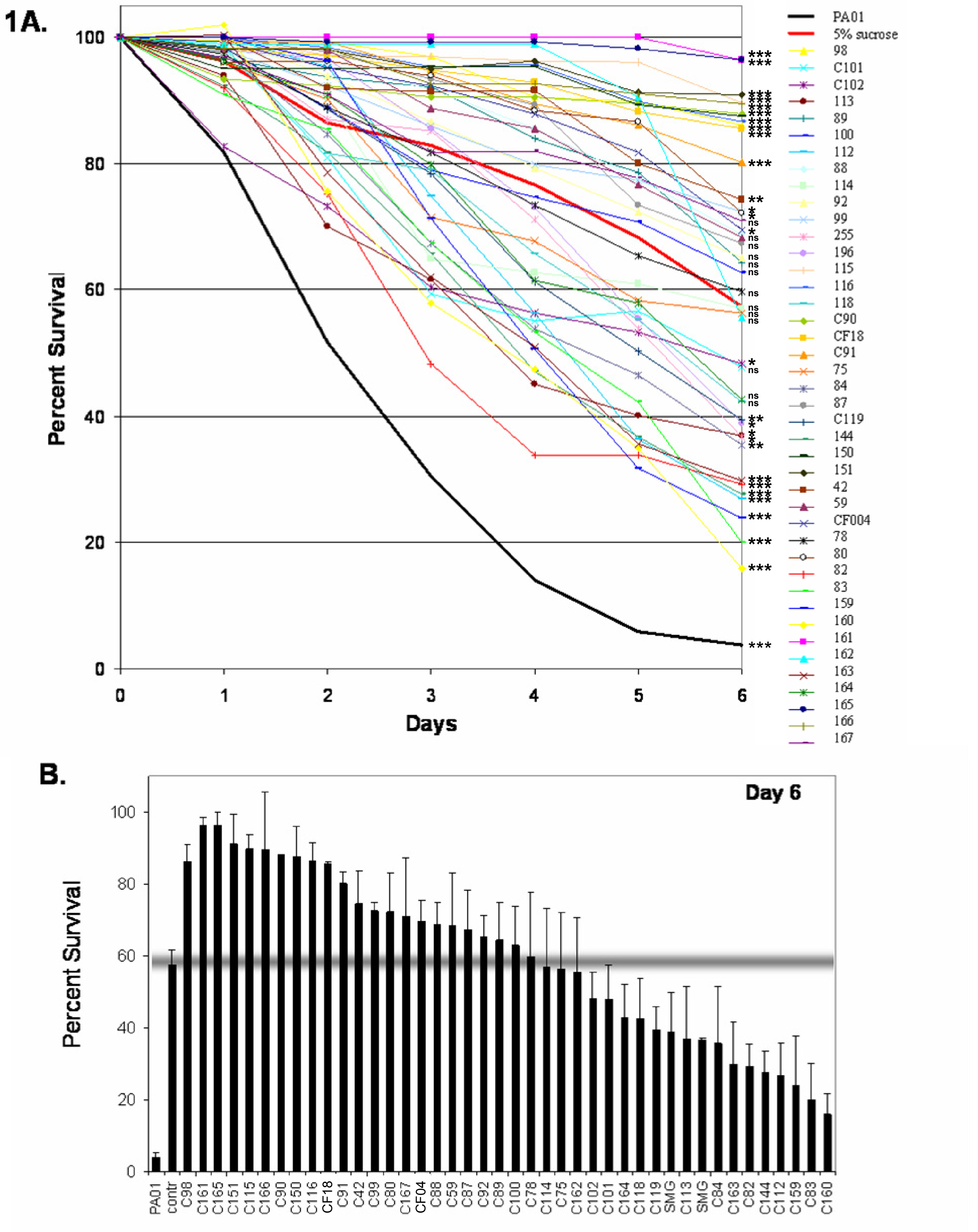
1A.
Percent Survival
Days
0
20
40
60
80
100
0
1
2
3
4
5
6
***
**
*
ns
PA01
5% sucrose
98
C101
C102
113
89
100
112
88
114
92
99
255
196
115
116
118
C90
CF18
C91
75
84
87
C119
144
150
151
42
59
CF004
78
80
82
83
159
160
161
162
163
164
165
166
167
B.
Day 6
Percent Survival
0
20
40
60
80
100
PA01
contr
C98
C161
C165
C151
C115
C166
C90
C150
C116
CF18
C91
C42
C99
C80
C167
CF04
C88
C59
C87
C92
C89
C100
C78
C114
C75
C162
C102
C101
C164
C118
C119
SMG
C113
SMG
C84
C163
C82
C144
C112
C159
C83
C160

Figure S1

Figure S1. A. Killing curves for single organism infections of *Drosophila.* The solid red line represents the negative control (5% sucrose) and represents the natural life span of the flies under our laboratory conditions. The infection with *P. aeruginosa* PAO1 is represented by the solid black line. The data was collected from at least six independent infections with a minimum of 25 flies per infection. Standard error was determined for each time point but is not included to simplify the figure. Log-rank analysis was used to compare the OF survival curves with the survival curve of the sucrose control; statistical significance between survival curves is shown beside the day 6 data point for each infection with * P <0.05, ** P <0.005 and *** P <0.0005; ns = not significant **B.** Percent survival on Day 6 of the infections showing the standard error for each infection. The *P. aeruginosa* and negative control (5% sucrose) are shown on the left. The infections with the OF strains are ordered by decreasing survival and standard error for each infection is indicated with the error bars. The broad gray line represents the negative control and infections above this have enhanced survival and those below represent virulent organisms.
