# Supplementary material for: Discerning the Complexity of Community Interactions Using a Drosophila Model of Polymicrobial Infections
Source: PLoS Pathog. 2008 Oct 24;4(10):e1000184. doi: 10.1371/journal.ppat.1000184 (PMC2566602; doi:10.1371/journal.ppat.1000184)
Supplement: Figure S2 — An example of a P. aeruginosa promoter unaffected in OF co-infections. (0.12 MB PDF) [file ppat.1000184.s004.pdf]

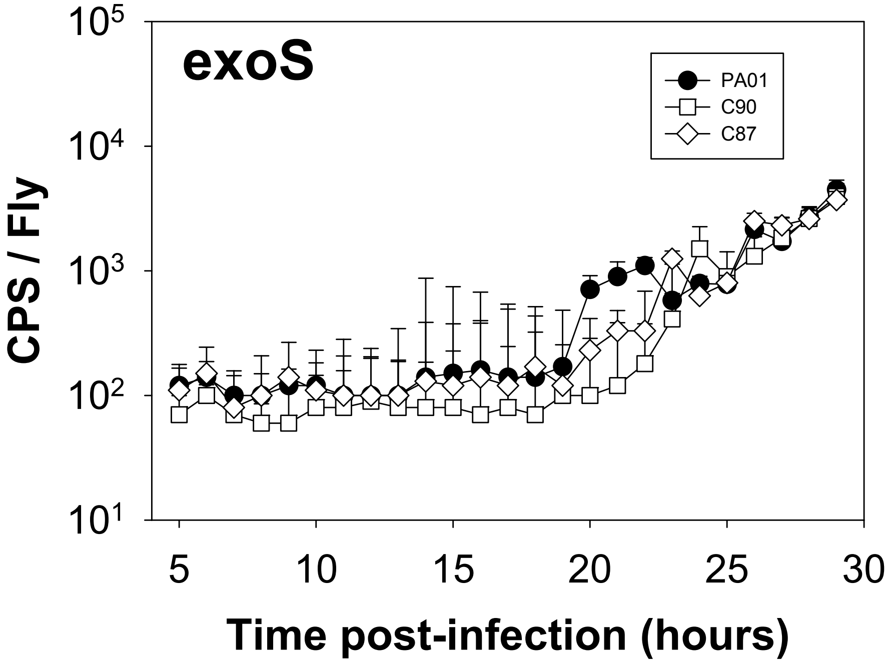

Figure S2. An example of a *P. aeruginosa* promoter unaffected in OF co-infections. The activity of the *P. aeruginosa exoS* promoter was measured every hour for 30 hours (in live flies). Expression levels are not significantly different between animals infected with *P. aeruginosa alone* (black circles) or co-infected with *P. aeruginosa* and an OF organism; C90 (open squares) or C87 (open diamonds).
